# Zika Virus Outbreak in Rio de Janeiro, Brazil: Clinical Characterization, Epidemiological and Virological Aspects

**DOI:** 10.1371/journal.pntd.0004636

**Published:** 2016-04-12

**Authors:** Patrícia Brasil, Guilherme Amaral Calvet, André Machado Siqueira, Mayumi Wakimoto, Patrícia Carvalho de Sequeira, Aline Nobre, Marcel de Souza Borges Quintana, Marco Cesar Lima de Mendonça, Otilia Lupi, Rogerio Valls de Souza, Carolina Romero, Heruza Zogbi, Clarisse da Silveira Bressan, Simone Sampaio Alves, Ricardo Lourenço-de-Oliveira, Rita Maria Ribeiro Nogueira, Marilia Sá Carvalho, Ana Maria Bispo de Filippis, Thomas Jaenisch

**Affiliations:** 1 Acute Febrile Illnesses Laboratory, Evandro Chagas National Institute of Infectious Diseases; Oswaldo Cruz Foundation (Fiocruz), Rio de Janeiro, Brazil; 2 Flavivirus Laboratory, Oswaldo Cruz Institute/ Oswaldo Cruz Foundation (Fiocruz), Rio de Janeiro, Brazil; 3 Scientific Computation Program, Oswaldo Cruz Foundation (Fiocruz), Rio de Janeiro, Brazil; 4 Clinical Research Platform, Evandro Chagas National Institute of Infectious Diseases; Oswaldo Cruz Foundation (Fiocruz), Rio de Janeiro, Brazil; 5 Mosquito Transmitters of Hematozoans Laboratory, Oswaldo Cruz Institute/ Oswaldo Cruz Foundation (Fiocruz), Rio de Janeiro, Brazil; 6 Section Clinical Tropical Medicine, Department of Infectious Diseases, Heidelberg University Hospital, Heidelberg, Germany & German Center for Infectious Disease Research, Heidelberg, Germany; Centers for Disease Control and Prevention, UNITED STATES

## Abstract

**Background:**

In 2015, Brazil was faced with the cocirculation of three arboviruses of major public health importance. The emergence of Zika virus (ZIKV) presents new challenges to both clinicians and public health authorities. Overlapping clinical features between diseases caused by ZIKV, Dengue (DENV) and Chikungunya (CHIKV) and the lack of validated serological assays for ZIKV make accurate diagnosis difficult.

**Methodology / Principal Findings:**

The outpatient service for acute febrile illnesses in Fiocruz initiated a syndromic clinical observational study in 2007 to capture unusual presentations of DENV infections. In January 2015, an increase of cases with exanthematic disease was observed. Trained physicians evaluated the patients using a detailed case report form that included clinical assessment and laboratory investigations. The laboratory diagnostic algorithm included assays for detection of ZIKV, CHIKV and DENV. 364 suspected cases of Zika virus disease were identified based on clinical criteria between January and July 2015. Of these, 262 (71.9%) were tested and 119 (45.4%) were confirmed by the detection of ZIKV RNA. All of the samples with sequence information available clustered within the Asian genotype.

**Conclusions / Significance:**

This is the first report of a ZIKV outbreak in the state of Rio de Janeiro, based on a large number of suspected (n = 364) and laboratory confirmed cases (n = 119). We were able to demonstrate that ZIKV was circulating in Rio de Janeiro as early as January 2015. The peak of the outbreak was documented in May/June 2015. More than half of the patients reported headache, arthralgia, myalgia, non-purulent conjunctivitis, and lower back pain, consistent with the case definition of suspected ZIKV disease issued by the Pan American Health Organization (PAHO). However, fever, when present, was low-intensity and short-termed. In our opinion, pruritus, the second most common clinical sign presented by the confirmed cases, should be added to the PAHO case definition, while fever could be given less emphasis. The emergence of ZIKV as a new pathogen for Brazil in 2015 underscores the need for clinical vigilance and strong epidemiological and laboratory surveillance.

## Author Summary

Zika virus (ZIKV) has been identified in 2015 in Brazil for the first time, causing outbreaks of an illness characterized by skin rash and absent or low grade and short-termed fever. It is difficult to distinguish ZIKV from Dengue (DENV) or (CHIKV) based on the acute clinical presentation. The virus is closely related to DENV, and therefore antibody tests also have problems distinguishing between the two viruses due to cross-reactivity. Recent findings suggest that in a minority of ZIKV cases neurological disease can develop, and that babies born from mothers reporting a ZIKV-like illness during pregnancy may suffer from congenital abnormalities, in many cases a small head or brain. Here we report about an outbreak of ZIKV disease in Rio de Janeiro in the first half of the year 2015, which reached its peak in May/June 2015. This is the first published description of a ZIKV outbreak in Latin America. It is interesting to note that confirmed cases appeared as early as January 2015. Cases were confirmed based on the detection of the viral genome in the blood of the patients. The clinical characterization of the confirmed cases and unconfirmed cases proved to be very similar. Itching or itching rash has been suggested to be added to the case definition issued by the Pan American Health Organization (PAHO).

## Introduction

### Background

Since 2015, Brazil is faced with the challenge of three co-circulating arboviruses of major public health importance. For the last 30 years, dengue virus (DENV) infection has been the main mosquito-transmitted threat in the country, which has suffered several epidemics caused by all four serotypes, [1, 2] fostered by the widespread presence of its main vector, *Aedes aegypti*, in densely populated urban areas. [3–5] In spite of all efforts, a sustained reduction of the *Ae aegypti* population has not been achieved.

The emergence of Chikungunya virus (CHIKV) and Zika virus (ZIKV) in Brazil [6, 7] poses new challenges to clinicians and public health authorities due to overlapping clinical features and the fact that validated serological assays for ZIKV that can reliably distinguish between acute disease and past exposure are currently not available. Most importantly, ZIKV infections are suspected to be associated with congenital abnormalities [8] and with neurological complications such as Guillan-Barré syndrome (GBS) [9], while CHIKV infections have been associated with persisting arthralgia [10].

Both ZIKV as well as DENV are members of the family *Flaviviridae*, whereas CHIKV is an alphavirus of the *Togaviridae* family. ZIKV was first described in humans in Africa in 1952 [11] and has recently caused epidemics in the Pacific region. [12–14] In May 2015, ZIKV was identified in Northeast Brazil associated with an outbreak of acute exanthematous disease in the state of Bahia, [6, 15] followed by several other locations, including the state of Rio de Janeiro. [6, 15–17] ZIKV was considered to cause a benign infection, leading to a self-limited disease consisting of rash, fever (often of low intensity and short-termed or even absent), and mild arthralgia and therefore did not receive much scientific attention until recently [14].

However, new findings suggest an association of recent ZIKV disease with GBS amongst adults[18] and with congenital abnormalities (e.g. microcephaly and ocular lesions in neonates) born from women reporting a ZIKV-like disease during pregnancy. [19–21]

Here we describe the clinical, epidemiological, and virological features of patients with acute exanthematous disease at a tertiary reference centre in Rio de Janeiro during an outbreak of ZIVK in the first half of 2015. We analyze the temporal and spatial occurrence of suspected ZIKV patients over the time of the outbreak and compare the profile of clinical signs and symptoms in PCR-confirmed ZIKV patients with the current PAHO case definition for ZIKV disease.

## Methods

### Setting

The study was conducted at the Laboratorio de Pesquisa Clínica de Doenças Febris Agudas of the Instituto Nacional de Infectologia Evandro Chagas (INI), which is part of the regional Dengue-Research Center of Fundação Oswaldo Cruz (FIOCRUZ), a reference center of the dengue network in Rio de Janeiro, Brazil. Patients with acute febrile disease seen at this outpatient clinic are either referred from other health units in Rio de Janeiro, or are spontaneously seeking care as they live in the nearby district of ‘Manguinhos’—an impoverished neighborhood close to the FIOCRUZ research center with a human development index of 0.726, a population of 36,160 persons in an area of 261 square kilometres, and an estimated number of 10,816 households.

Since January 2015 physicians at INI/FIOCRUZ observed an increase of cases characterized by rash, with either absent or low-grade and short-termed fever, and sometimes associated with arthralgia and/or conjunctivitis. The disease was clinically distinct from DENV which led to a systematic syndromic investigation utilizing a specific laboratory algorithm, which included diagnostics assays for detection of DENV, CHIKV, and ZIKV (after the reports of ZIKV transmission in the Northeast of Brazil[22]). The investigations were performed as part of the ongoing study on “Detection of unusual clinical presentations of dengue”, which was reviewed and approved by the local Ethics Committee (CAAE 0026.0.009.000–07). Written informed consent was obtained from all patients or their legal representatives.

For the purpose of the analysis presented here we concentrate on the first half of the year 2015. After the identification of ZIKV infection in a patient with no travel history outside Rio de Janeiro in May 2015[16], additional prospective surveillance at the outpatients' clinic was initiated in order to specifically identify patients with suspected ZIKV disease. Patients with acute onset of generalized macular or papular rash were considered suspected cases of ZIKV. We interviewed patients using a standard case report form (CRF) to collect information about demographic features, clinical signs and symptoms, and the severity of the disease (S1 File). Blood samples were collected during the acute phase (i.e., within 7 days after the onset of symptoms) and during the convalescent phase. Samples were stored at -70°C and analyzed in the Flavivirus Diagnostic and Reference Laboratory of Fiocruz.

### Laboratory Analysis and Confirmation

Acute phase serum samples were tested by qRT-PCR for ZIKV [23] and RT-PCR for DENV RNA [24]. CHIKV qRT-PCR testing was performed for a random sample of 25% of patients as no patient tested positive and there was no on-going transmission of CHIKV in Rio at the time of this stud1y. Cases were classified as ZIKV infection if ZIKV RNA was detected in the serum.

### Phylogenetic Analysis

Phylogenetic analysis of nucleic acid sequences derived from 10 random ZIKV positive samples (out of 119) was performed using 327 base pairs of the envelope protein (GeneBank accession number KT381874). The tree was inferred using the maximum likelihood algorithm based on the Tamura 3-parameter model as implemented in MEGA 6. The numbers shown to the left of the nodes represent bootstrap support values > 70 (1,000 replicates). The tree was rooted with Spondoweni virus.

### Statistical Analysis

The home addresses of the cases were georeferenced using Google maps. Statistical analyses and maps were performed using software R version 3.2.2.libraries RCurl, RJSONIO, ggmap and ggplot2. [25]

## Results

**Fig 1:**
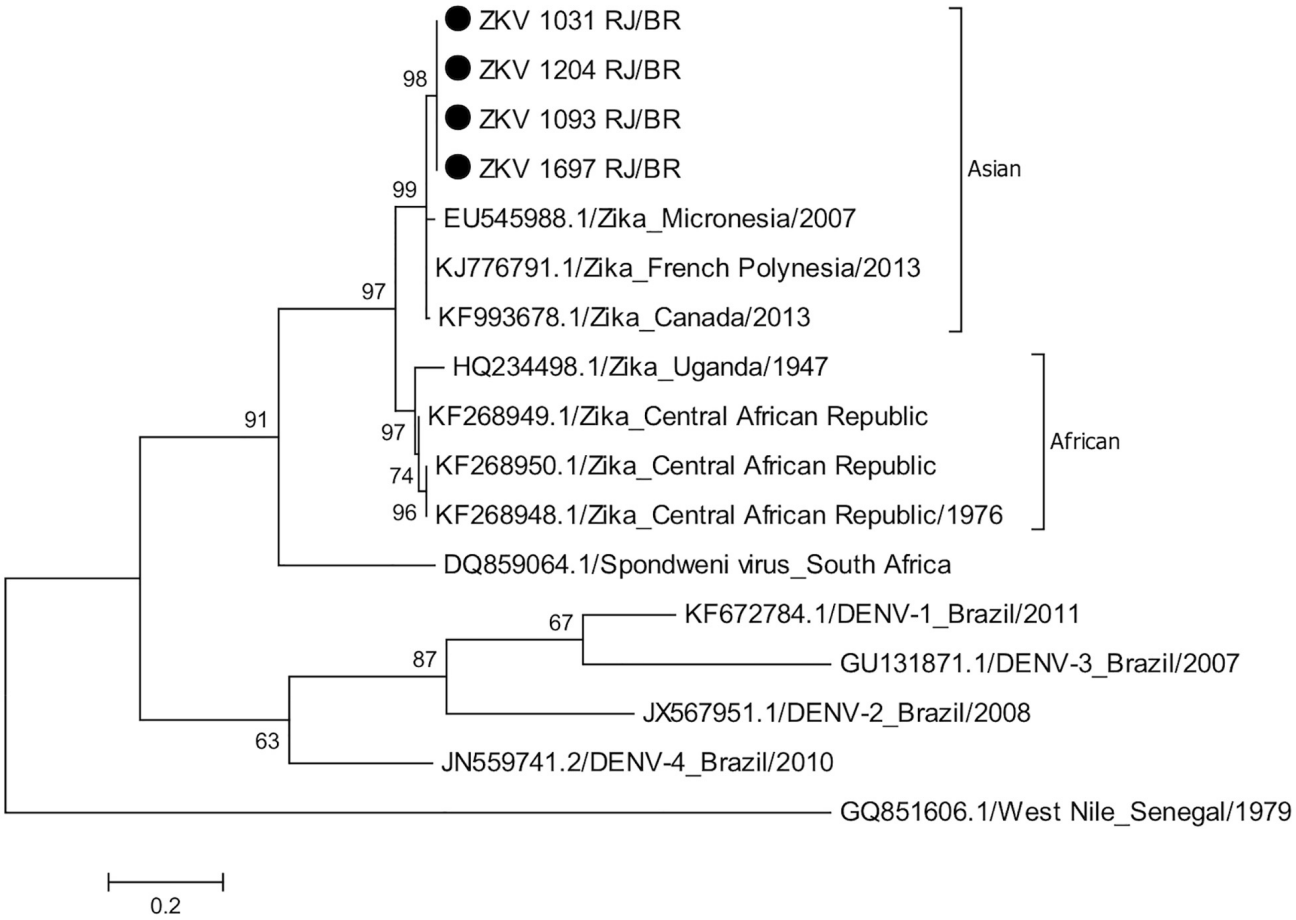
Phylogenetic analysis based on partial E gene nucleic acid sequences (327bp). Closed circles represent 4 strains of Zika virus from Rio de Janeiro, Brazil 2015. The remaining 6 strains were identical to these 4 and are not shown here. The tree was inferred using the maximum likelihood algorithm based on the Tamura 3-parameter model as implemented in MEGA 6. The numbers shown to the left of the nodes represent bootstrap support values > 70 (1,000 replicates). The tree was rooted with West Nile virus.

Between 1^st^ of January and 31^st^ of July 2015, 364 suspected cases of ZIKV disease were identified based on the presence of acute onset rash with or without fever. Of these, 262 (71.9%) were tested and 119 (45.4%) were confirmed by the detection of ZIKV RNA through qRT-PCR assay. All of the samples with sequence information available clustered within the Asian genotype (Fig 1). RT-PCR assays using consensus primers for nucleic acid of other arboviruses, including DENV and CHIKV, were negative in these patients. No DENV RNA was detected in any of the 143 acute phase serum samples that tested negative for ZIKV.

**Fig 2:**
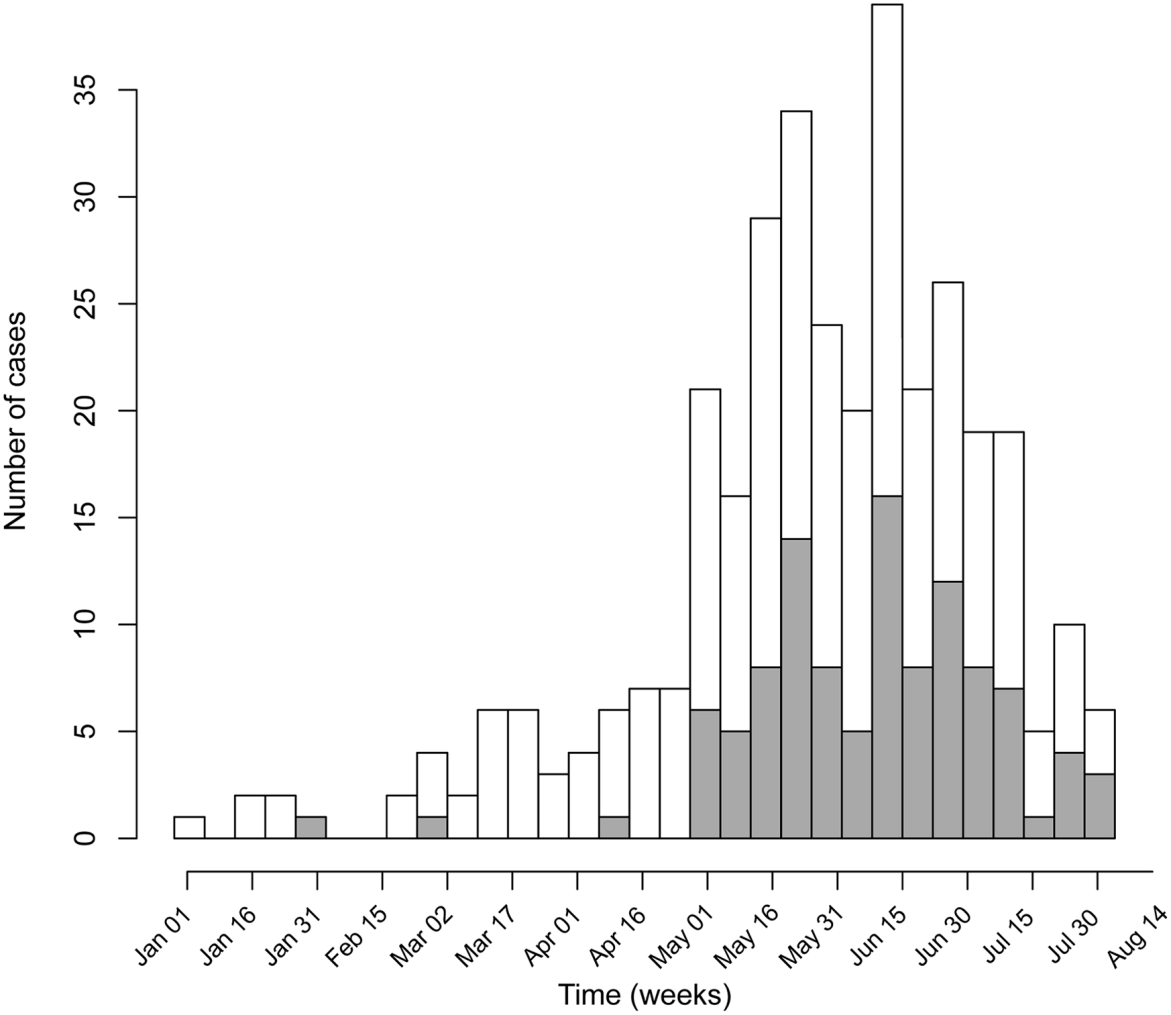
Time series for number of cases confirmed (gray background) and not confirmed (white background) for ZIKV between January 1, 2015 and July 31, 2015 in Rio de Janeiro State.

**Fig 3:**
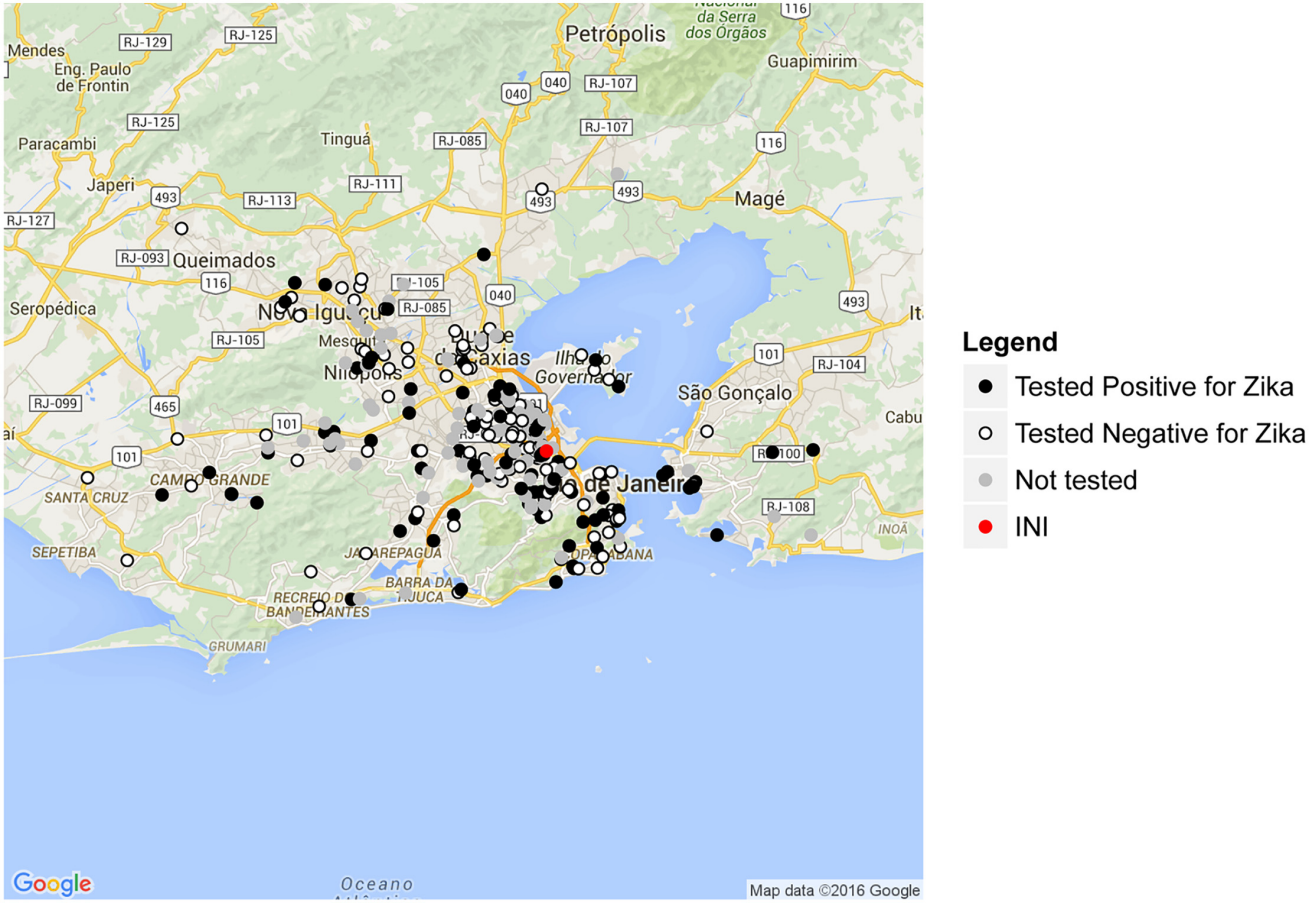
Spatial distribution at Rio de Janeiro State for cases tested positive (black dots), tested negative (dark gray dots) and not tested yet (light gray) for ZIKV between January 1, 2015 and July 31, 2015. The red dot indicates the Instituto Nacional de Infectologia, where patients were seen.

Suspected ZIKV cases started to appear in January 2015, peaking in May (Fig 2). Their geographical distribution is shown in Fig 3. In spite of the clustering in the surrounding neighborhood ‘Manguinhos’, cases residing in other neighborhoods in the Metropolitan area of Rio de Janeiro were also included (Fig 3).

Baseline characteristics of confirmed (tested ZIKV positive) and unconfirmed (tested ZIKV negative) patients are presented in Table 1. The median age of these patients was 37 years (range 9 to 60). A majority of the cases were females (158/262–60.3%), and among these 73% were aged 15–49 years (116/158–73.4%). The same pattern was seen with regard to the confirmed cases only (60.5% female [72/119] and 76.4% among those in the age group of 15–49 years [55/72]). No travel histories were recorded for the confirmed cases, thus infections were acquired locally. Only 38% of the patients recalled exposure to mosquito bites. Arterial hypertension was the most frequent comorbidity recorded, followed by diabetes. Geographic clustering of cases (defined as more than one case in the household, neighborhood or work) occurred in half of all confirmed cases (62/119–51.1%).

**Table 1:** Baseline characteristics of confirmed and unconfirmed ZIKV disease patients.

Patients Characteristics	Confirmed (n = 119) n(%)	Unconfirmed* (n = 143) n(%)	Total (n = 262) n(%)
**Gender**			
Male	47(40)	57(40)	104(40)
Female	72(61)	86(60)	158(60)
**Age group**			
8–14	4(3)	6(4)	10(4)
15–19	7(6)	6(4)	13(5)
20–29	28(24)	29(20)	57(22)
30–39	31(26)	43(30)	74(29)
40–49	25(21)	25(18)	50(19)
50–59	18(15)	21(15)	39(15)
> = 60	6(5)	13(9.)	19(7)
**Comorbidities**			
Hypertension	18(15)	20(14)	38(15)
Diabetes mellitus	6(7)	14(10)	20(8)
Asthma	4(3)	4(3)	8(3)
Smoking	11(9)	13(9)	24(9)
Regular alcohol comsumption	38(32)	36(25)	74(28)
Rhinitis	20(17)	21(15)	41(16)
**Clustering**	**62(52)**	**65(46)**	**127(49)**
Household	28(45)	25(39)	53(42)
Neighborhood	20(32)	19(29)	39(31)
Work	14(23)	21(32)	35(28)
**Insect bite**	**48(40)**	**50(35)**	**98(38)**

* tested ZIKV negative

The most commonly reported symptoms in the first four days of the disease were rash, itching, prostration, headache and arthralgia (with or without associated oedema) (Table 2). Although fever was not observed at presentation in the majority of patients (64%), 43 patients (36%) reported a history of fever not lasting longer than one day, or the occurrence of a single fever peak on the first day of disease. The median duration of rash was 5.5 days (range 3 to 7), and of arthralgia 9 days (range 2 to 21). Mild hemorrhagic symptoms (petechiae, minor mucosal bleeding) were reported in 21% of the confirmed and 11% of the unconfirmed cases, while no severe bleeding was observed. One patient was hospitalized with neurological symptoms consisting of peripheral nerve impairment, and is described in more detail elsewhere (Brasil et al, in press). No deaths or other severe complications were associated with the ZIKV disease in this series. Four women with confirmed ZIKV were pregnant. One had a miscarriage at 10 weeks of pregnancy, three weeks after the acute disease episode. The remaining three (two with ZIKV infection during the 18^th^ week and one during the 35^th^ week) delivered normal babies without any clinical evidence of infection or congenital abnormalities.

**Table 2:** Clinical characteristics of confirmed and unconfirmed* ZIKV infected patients, in the first four days of disease.

Sign/Symptom	Confirmed n(%)	Unconfirmed n(%)
Macular or papular rash	115(97)	113(79)
Itching	94(79)	127(89)
Prostration	87(73)	104(73)
Headache	78(66)	101(71)
Arthralgia	75(63)	105(73)
Myalgia	73(61)	96(67)
Nonpurulent conjuntivitis	66(56)	57(40)
Low back pain	61(51)	70(49)
Retro-orbital pain	53(45)	76(53)
Lymphonodes elargement	49(41)	33(23)
Chills	44(37)	52(36)
Fever	43(36)	71(50)
Anorexia	42(35)	60(42)
Photophobia	41(345)	43(30)
Oropharyngeal pain	38(32)	35(25)
Edema	34(29)	26(18)
Taste alteration	32(27)	33(23)
Nausea	28(24)	43(30)
Enanthema/Petechiae/Bleeding	25(21)	16(11)
Nasal congestion	24(20)	25(18)
Sweating	23(19)	38(27)
Diarrhea	23(19)	21(15)
Abdominal pain	20(17)	29(20)
Cough	19(16)	28(20)
Coryza	18(15)	20(14)
Lipothymia	18(15)	28(20)
Hoarseness	13(11)	13(9)
Earache	11(9)	8(5)
Dysuria	8(7)	5(4)
Choluria	7(6)	8(6)
Dyspnea	7(6)	10(7)
Vomiting	5(4)	10(7)
Hepatomegaly	2(2)	2(1)

* tested ZIKV negative

All patients had full blood counts performed. The median leucocyte count of confirmed ZIKV cases was 4,590 cells/mm^3^ (2,240 to 11,570 cells/mm^3^), the median platelet count was 201,000 cells/mm^3^ (102,000 to 463,000 cells/mm^3^) and the median haematocrit was 41.2% (33.2 to 50.3%).

## Discussion

This is the first report of a ZIKV outbreak in the state of Rio de Janeiro, based on a large number of suspected (n = 364) and laboratory confirmed cases (n = 119). We were able to demonstrate that ZIKV was circulating in Rio de Janeiro as early as January 2015. The outbreak began in the first half of 2015 with a transmission peak in May 2015. The clinical picture of the disease was characterized by the sudden increase of exanthematous disease with absent or short-termed and low-grade fever.

The early documentation as well as the detailed clinical characterization was possible through a prospective syndromic surveillance study, which included a systematic collection of blood samples for the detection of unusual forms of dengue. In fact, 11% of the confirmed cases date back to the months before ZIKV transmission was reported in Northeast Brazil, in May (Fig 2).

The detection of ZIKV RNA in the serum of acutely ill patients and the absence of nucleic acid of other arboviruses provide convincing evidence that the outbreak was caused by ZIKV. Although DENV is common in Rio de Janeiro, none of the 252 patients for whom acute-phase specimens were available, tested positive for DENV, which is surprising and possibly highlights explosive transmission dynamics of ZIKV. Patients were also tested for other pathogens at the attending physician’s discretion, but no other causes were found (i.e. CHIKV, rubella, cytomegalovirus, EBV, toxoplasmosis). Based on this study, there is no evidence that ZIKV was circulating in Rio de Janeiro before January 2015. However, a larger and more representative sample would be necessary to make a more definitive statement about ZIKV circulation in Rio de Janeiro before 2015.

The peak of cases was observed in May (Fig 2), after the rainy season—which is in line with temporal patterns observed in previous outbreaks in Yap Island and French Polynesia. [13, 14] In May 2015, atypically high precipitation was recorded in Rio de Janeiro [26], which may have affected the vector distribution and abundance.

It is noteworthy, that the first DENV epidemic in Rio de Janeiro in 1986 started in the same geographical area from where the majority of ZIKV cases reported here originated [27]. This is likely to be associated with the high human population density, abundant *Ae aegypti* populations, the precarious socioeconomic status, and lack of infrastructure.

The observation that ZIKV cases clustered within households is interesting and needs to be verified in other settings. If this is a consistent feature, it could be consistent with a high vectorial capacity or hint towards other routes of transmission. The possibility of transmission via mucosal contact is supported by evidence of sexual transmission [28, 29] and by the detection of ZIKV RNA in saliva and urine. [30, 31] Further studies need to be carried out to address this question in more detail, with important implications on epidemiology and disease control of ZIKV.

The age distribution with a bias towards adults is consistent with the overall age profile of the clinic. Female patients were overrepresented with regard to males, which might be due to differences in health care–seeking behavior.

An epidemic of microcephaly in newborns in Northeast Brazil has been the focus of attention by public health authorities in Brazil and worldwide. There is emerging evidence that these congenital abnormalities may be associated with exposure to ZIKV during pregnancy. [32] Among the ZIKV-positive pregnant women followed in our study, one out of four had a miscarriage in the 10^th^ week of pregnancy. However, no further investigations were performed in this case. In a recently published cohort of pregnant women with ZIKV infection, we have described two miscarriages amongst 72 confirmed cases [21], although the real frequency of this and other complications is yet to be determined. More recently, the state of Rio de Janeiro has reported a tenfold increase in microcephaly cases in 2015 compared to previous years [32] and, due to the long-lasting medical and economic consequences, it is of high importance that multidisciplinary studies are conducted to determine the incidence and the phenotypic variability of congenital abnormalities related to ZIKV infection during pregnancy.

The clinical signs and symptoms of ZIKV observed in Rio de Janeiro have many similarities with those described in previous reports and case series. [14] The similarity and frequency of clinical manifestations between confirmed and unconfirmed cases supports the notion that the majority of suspect cases were suffering from ZIKV disease, which could be due to current limits of detecting low quantities of RNA within a potentially short viremic period and the lack of a reliable serological test to ascertain recent exposure and distinguish it from DENV infections.

As in DENV and CHIKV infections, rash was a common feature in ZIKV. However, pruritus/itching was a prominent characteristic of the maculo-papular rash in the majority of confirmed cases (79%) from the onset of symptoms, which can potentially help in distinguishing ZIKV from other arboviral infections. Enlarged lymph nodes, especially in the cervical and retro-auricular regions, were also found frequently, which led clinicians to consider rubella as an important differential diagnosis. More than half of the patients reported headache, arthralgia, myalgia, non-purulent conjunctivitis, and lower back pain, consistent with the case definition of suspected ZIKV issued by PAHO. [8] However, fever was absent in most cases. In our opinion, pruritus, the second most common clinical sign presented by the confirmed cases, should be added to the PAHO case definition.

DENV, which has been circulating in Brazil for many years, was considered an unlikely diagnosis in many patients because fever was only observed in a minority (36%). When present, fever was usually short-termed and the temperature relatively low. From a clinical management perspective, it is important to differentiate ZIKV both from CHIKV and especially from DENV due to the need to monitor cases for possible evolution of severe and life-threatening clinical outcomes. Standard laboratory values (e.g. hematology or biochemistry) did not show a distinct pattern in the confirmed ZIKV patients and are unlikely to be helpful. The leucocyte count in ZIKV patients was in many cases moderately decreased as in other viral infections (< 5,000 cells mm^3^) and both platelet counts and haematocrit were within the normal range.

In this study, only one patient was hospitalized with febrile illness and a neurological manifestation developed afterwards (manuscript submitted). An increase in cases with Gullain-Barré syndrome (GBS) was reported during the epidemic In French Polynesia in 2013/14 [12], as well as in the Northeast of Brazil during the current outbreak. [6] It remains to be seen if ZIKV is causally related to higher rates of neurological complications.

Our data suggests that in a previously naïve adult population, ZIKV causes a self-limited and mostly benign disease characterized by a pruritic maculopapular rash and absent or low-grade, short-termed fever. ZIKV-like disease was not a notifiable condition in Rio de Janeiro until October 22th, 2015. This partially explains why our data differs from the official figures, [22] as the syndromic surveillance study that was implemented in 2007 (long before the ZIKV outbreak started) is more likely to detect changes in the clinical patterns of dengue compared to the routine health system. Similar to the dynamic in the Northeast of Brazil, a considerable increase in the number of microcephaly in neonates was registered in Rio de Janeiro during 2015. As of January 2016, 122 microcephaly cases were documented—compared to the average of 10 cases per year registered in the previous years. [32] This increase could potentially be attributed to the first wave of ZIKV transmission observed between January and July 2015 in Rio de Janeiro. In Pernambuco, in the Northeast region of Brazil, 1306 cases of microcephaly were reported as of the second epidemiological week of 2016 [32] which are attributed to the ZIKV outbreak in early spring 2015.

At this point, health services must be alerted to the potential for an even larger epidemic during the summer of 2015–2016 spreading to additional locations and affecting the susceptible proportion of the population that was not exposed during the last transmission season.

The emergence of ZIKV as a new pathogen for Brazil in 2015 underscores the ease with which pathogens travel between continents and the need for clinical vigilance and strong epidemiological and laboratory surveillance systems. The phylogenetic analysis somehow is in line with the speculative hypothesis that ZIKV was possibly introduced to Rio de Janeiro during the VI World Sprint Championship canoe race in August 2014, which included teams from four Pacific countries (French Polynesia, New Caledonia, Cook Islands, and Easter Island) where the virus circulated during 2014. [33]

In 2016, Rio de Janeiro will be hosting the Summer Olympics and Paralympics games, which will attract a high number of national and international visitors. The threat caused by ZIKV has far reaching implications on tourism and industry.[34] Reliable and sensitive surveillance of arboviral disease that includes a system for the detection of emerging pathogens is of paramount importance to manage the complex challenges ahead. Our findings have demonstrated that ZIKV was circulating in Rio de Janeiro at least five months before its detection was announced by the health authorities, which must be taken into consideration for future design and implementation of effective syndromic surveillance systems.

## Supporting Information

S1 File Standard case report form. (PDF)
