# Supplementary material for: Zika Virus Outbreak in Rio de Janeiro, Brazil: Clinical Characterization, Epidemiological and Virological Aspects
Source: PLoS Negl Trop Dis. 2016 Apr 12;10(4):e0004636. doi: 10.1371/journal.pntd.0004636 (PMC4829157; doi:10.1371/journal.pntd.0004636)
Supplement: S1 File — (PDF) [file pntd.0004636.s001.pdf]

2797030031

FIOCRUZ

Código/BE

Ambulatório Sentinela de Doenças Febris Agudas / Dengue (IPEC-Fiocruz)

Cod

Observador

☐ 1 Patricia ☐ 4 Rogério
☐ 2 Clarisse ☐ 5 Edwiges
☐ 3 Carolina ☐ 6 outro

☐ **1 A**
☐ **2 B**

Data de Atendimento __/__/____

Nome

Data de Nascimento __/__/____

Cor
☐ 1 Branca ☐ 3 Amarelo
☐ 2 Preta ☐ 4 Parda
☐ 5 Indio

Grau de Instrução
☐ 1 Analfabeto ☐ 5 2º Grau Completo
☐ 2 1º Grau Incompleto ☐ 6 3º Grau Incompleto
☐ 3 1º Grau Completo ☐ 7 3º Grau Completo
☐ 4 2º Grau Incompleto ☐ 9 SD

Nat

Sexo
☐ 1 M ☐ 2 F

Nome da Mãe

Endereço Residência

Bairro

Tempo de residência (em anos)

Localidade
☐ 1 Rural ☐ 2 Urbana

Cidade

Estado

Telefone ____-____

Telefone contato ____-____

Ocupação

Bairro de Trabalho

Dados epidemiológicos (últimos 30 dias)

Viagem Recente
☐ 1 Sim ☐ 2 Não

Data da viagem __/__/____ a __/__/____

Local

Casos semelhantes
☐ 1 Sim ☐ 2 Não

Casa
☐ 1 Sim ☐ 2 Não

Vizinho
☐ 1 Sim ☐ 2 Não

Trabalho
☐ 1 Sim ☐ 2 Não

Exposição Enchente
☐ 1 Sim ☐ 2 Não

Entrada em vala negra
☐ 1 Sim ☐ 2 Não

Entrada em cavernas
☐ 1 Sim ☐ 2 Não

Limpeza de Esgotos/Fossa
☐ 1 Sim ☐ 2 Não

Limpeza de caixa D'água
☐ 1 Sim ☐ 2 Não

Hemotransfusão
☐ 1 Sim ☐ 2 Não

Limpeza de locais c/ ratos
☐ 1 Sim ☐ 2 Não

Área de Malária
Último 3 meses
☐ 1 Sim ☐ 2 Não

Contato c/carrapatos
☐ 1 Sim ☐ 2 Não

Contato com animais doentes
☐ 1 Sim ☐ 2 Não

Contato com Caramujo
☐ 1 Sim ☐ 2 Não

Contato com aves
☐ 1 Sim ☐ 2 Não

Ingestão de peixe cru
☐ 1 Sim ☐ 2 Não

Ingestão de leite cru
☐ 1 Sim ☐ 2 Não

Ingestão de Açaí
☐ 1 Sim ☐ 2 Não

Abastecimento de água:
☐ 1 Rede geral ☐ 2 Poço/Nascente
☐ 3 Outra

Tratamento da água de beber:
☐ 1 não ☐ 2 clora/filtra/ferve
☐ 3 água mineral

Cod

0404030034

Contato c/ carcaça placenta de animais
☐ 1 Sim ☐ 2 Não

Gestante
☐ 1 Sim ☐ 2 Não

Picada de Inseto
☐ 1 Sim ☐ 2 Não

Inseto

Histórico de Dengue
☐ 1 Sim ☐ 2 Não
Ano

Vacina febre amarela (Ultimos 10 anos)
☐ 1 Sim ☐ 2 Não

Quimioprofilaxia malária
☐ 1 Sim ☐ 2 Não

Qual? ____________________

Período: ____________________

Vacina rubeola
☐ 1 Sim ☐ 2 Não

Comorbidades:

Diabetes Mellitus
☐ 1 Sim ☐ 2 Não

Rinite
☐ 1 Sim ☐ 2 Não

DPOC
☐ 1 Sim ☐ 2 Não

Uso de alcool
☐ 1 Sim ☐ 2 Não

Hipertensão Arterial
☐ 1 Sim ☐ 2 Não

Uso de tabaco
☐ 1 Sim ☐ 2 Não

Asma Bronquica
☐ 1 Sim ☐ 2 Não

Anemia falciforme
☐ 1 Sim ☐ 2 Não

Dç imunossupressoras
☐ 1 Sim ☐ 2 Não

Tx de órgãos/MO
☐ 1 Sim ☐ 2 Não

Uso de medicamentos
☐ 1 sim ☐ 2 nao

Qual medicamento?
☐ 1 Analgesicos / antitermicos
☐ 2 Anti-inflamatorios nao esteroides
☐ 3 Anti-hipertensivos
☐ 4 Hipoglicemiantes orais
☐ 5 Corticosteroides (sistemicos)
☐ 6 Antimicrobianos
☐ 7 outros

Drogas ilícitas
☐ 1 Sim ☐ 2 Não

Uso de drogas injetáveis
☐ 1 Sim ☐ 2 Não

Qual droga
☐ 1 depressores
☐ 2 estimulantes
☐ 3 alucinogenos
☐ 4 multiplas

Tabela I (Dados Clínicos)

Queixa Principal

Data dos Primeiros Sintomas
__ / __ / ____

Data do aparecimento da febre
__ / __ / ____

Data do desaparecimento da febre
__ / __ / ____

Maior temperatura aferida

☐ (0) ausente	☐ ausente
☐ (1) 37,1 -37,5	☐ 37,1 -37,5
☐ (2) 37,6 - 38,5	☐ 37,6 - 38,5
☐ (3)> 38,5	☐ > 38,5
☐ (4) nao aferida	☐ nao aferida

Início
☐ 1 Subito
☐ 2 Gradual

Evolucao
☐ 1 contínua
☐ 2 Intermitente, irregular
☐ 3 Intermitente,vespertina
☐ 4 Intermitente, tercã
☐ 5 Intermitente, quartã

Data do primeiro
__ / __ / ____

Data exame
__ / __ / ____

**Calafrios**	**Cefaléia**	**Fotofobia**	**Dor retrorbital**	**Dor em orofaringe**
☐ 0 ☐ 0	☐ 0 ☐ 0	☐ 0 ☐ 0	☐ 0 ☐ 0	☐ 0 ☐ 0
☐ 1 ☐ 1	☐ 1 ☐ 1	☐ 1 ☐ 1	☐ 1 ☐ 1	☐ 1 ☐ 1
☐ 2 ☐ 2	☐ 2 ☐ 2	☐ 2 ☐ 2	☐ 2 ☐ 2	☐ 2 ☐ 2
☐ 3 ☐ 3	☐ 3 ☐ 3	☐ 3 ☐ 3	☐ 3 ☐ 3	☐ 3 ☐ 3

Rouquidão
☐ 0 ☐ 0
☐ 1 ☐ 1
☐ 2 ☐ 2
☐ 3 ☐ 3

Alteração de Paladar
☐ 1 Sim ☐ 1 Sim
☐ 2 Não ☐ 2 Não
Qual:

Dor de Ouvido
☐ 0 ☐ 0
☐ 1 ☐ 1
☐ 2 ☐ 2
☐ 3 ☐ 3

Coriza
☐ 0 ☐ 0
☐ 1 ☐ 1
☐ 2 ☐ 2
☐ 3 ☐ 3

Congestão Nasal
☐ Sim ☐ Sim
☐ Não ☐ Não

Sudorese
☐ Sim ☐ Sim
☐ Não ☐ Não

5323030039

Cod

Prostração
- ☐ 0 ☐ 0
- ☐ 1 ☐ 1
- ☐ 2 ☐ 2
- ☐ 3 ☐ 3

Mialgia
- ☐ 0 ☐ 0
- ☐ 1 ☐ 1
- ☐ 2 ☐ 2
- ☐ 3 ☐ 3

Lombalgia
- ☐ 0 ☐ 0
- ☐ 1 ☐ 1
- ☐ 2 ☐ 2
- ☐ 3 ☐ 3

Artralgia
- ☐ 0 ☐ 0
- ☐ 1 ☐ 1
- ☐ 2 ☐ 2
- ☐ 3 ☐ 3

Anorexia
- ☐ 0 ☐ 0
- ☐ 1 ☐ 1
- ☐ 2 ☐ 2
- ☐ 3 ☐ 3

Náuseas
- ☐ 0 ☐ 0
- ☐ 1 ☐ 1
- ☐ 2 ☐ 2
- ☐ 3 ☐ 3

Vômitos
- ☐ 0 ☐ 0
- ☐ 1 ☐ 1
- ☐ 2 ☐ 2
- ☐ 3 ☐ 3

Dor Abdominal
- ☐ 0 ☐ 0
- ☐ 1 ☐ 1
- ☐ 2 ☐ 2
- ☐ 3 ☐ 3

Colúria
- ☐ 1 Sim ☐ Sim
- ☐ 2 Não ☐ Não

Disúria
- ☐ 1 Sim ☐ Sim
- ☐ 2 Não ☐ Não

Diarréia
- ☐ 1 Sim ☐ 1 Sim
- ☐ 2 Não ☐ 2 Não

Nº de evacuações / Dia

Consistência
- ☐ 1 Liq ☐ Liq
- ☐ 2 Past. ☐ Past.

Sangue
- ☐ 1 Sim
- ☐ 2 Não

Muco
- ☐ 1 Sim
- ☐ 2 Não

Dispnéia
- ☐ 0 ☐ 0
- ☐ 1 ☐ 1
- ☐ 2 ☐ 2
- ☐ 3 ☐ 3

Tosse
- ☐ 0 ☐ 0
- ☐ 1 ☐ 1
- ☐ 2 ☐ 2
- ☐ 3 ☐ 3

Tosse Produtiva
- ☐ 0 ☐ 0
- ☐ 1 ☐ 1
- ☐ 2 ☐ 2
- ☐ 3 ☐ 3

Hemorragias
- ☐ 1 Sim ☐ 1 Sim
- ☐ 2 Não ☐ 2 Não

Epistaxe
- ☐ 0 ☐ 0
- ☐ 1 ☐ 1
- ☐ 2 ☐ 2
- ☐ 3 ☐ 3

Gengivorragia
- ☐ 0 ☐ 0
- ☐ 1 ☐ 1
- ☐ 2 ☐ 2
- ☐ 3 ☐ 3

Metrorragia
- ☐ 0 ☐ 0
- ☐ 1 ☐ 1
- ☐ 2 ☐ 2
- ☐ 3 ☐ 3

Hematúria
- ☐ 0 ☐ 0
- ☐ 1 ☐ 1
- ☐ 2 ☐ 2
- ☐ 3 ☐ 3

Hematêmese
- ☐ 0 ☐ 0
- ☐ 1 ☐ 1
- ☐ 2 ☐ 2
- ☐ 3 ☐ 3

Melena
- ☐ 1Sim ☐ 1 Sim
- ☐ 2 Não ☐ 2 Não

Prurido
- ☐ 0 ☐ 0
- ☐ 1 ☐ 1
- ☐ 2 ☐ 2
- ☐ 3 ☐ 3

Hemoptóicos / Hemoptise
- ☐ 0 ☐ 0
- ☐ 1 ☐ 1
- ☐ 2 ☐ 2
- ☐ 3 ☐ 3

Lipotímia
- ☐ 0 ☐ 0
- ☐ 1 ☐ 1
- ☐ 2 ☐ 2
- ☐ 3 ☐ 3

Exantema
- ☐ 0 ☐ 0
- ☐ 1 ☐ 1
- ☐ 2 ☐ 2
- ☐ 3 ☐ 3

Convulsões
- ☐ 0 ☐ 0
- ☐ 1 ☐ 1
- ☐ 2 ☐ 2
- ☐ 3 ☐ 3

7663030038

Exame Físico :

Cod ☐☐☐☐

Data do primeiro atendimento ☐☐ / ☐☐ / ☐☐☐☐

Data 2º Atendimento ☐☐ / ☐☐ / ☐☐☐☐

Assinale 0 - Ausente / 1 - Leve / 2 - Moderado / 3 - Grave ou Sim Não, exceto quando a resposta for numérica (Temp ax, PA, etc)

Temp Ax(ºC) ☐☐,☐ Temp Ax(ºC) ☐☐,☐

Freq. cardíaca ☐☐☐ Freq. cardíaca ☐☐☐

Freq. Resp. ☐☐ Freq. Resp. ☐☐

Peso (kg) ☐☐☐,☐ Peso (kg) ☐☐☐,☐

PA sentada ☐☐☐ / ☐☐☐ PA ☐☐☐ / ☐☐☐

PA deitada ☐☐☐ / ☐☐☐ PA ☐☐☐ / ☐☐☐

Enchimento capilar lentificado
☐ 1 Sim ☐ 1 Sim
☐ 2 Não ☐ 2 Não

Pulso filiforme
☐ 1 Sim ☐ 1 Sim
☐ 2 Não ☐ 2 Não

Extremidades frias	Hipotensão/Choque	Dispnéia	Desidratação
☐ 1 Sim ☐ 1 Sim<br>☐ 2 Não ☐ 2 Não	☐ 1 Sim ☐ 1 Sim<br>☐ 2 Não ☐ 2 Não	☐ 0 ☐ 0<br>☐ 1 ☐ 1<br>☐ 2 ☐ 2<br>☐ 3 ☐ 3	☐ 0 ☐ 0<br>☐ 1 ☐ 1<br>☐ 2 ☐ 2<br>☐ 3 ☐ 3
**Palidez cutânea mucosa**	**Edemas**	**Congestão ocular**	**Icterícia**
☐ 0 ☐ 0<br>☐ 1 ☐ 1<br>☐ 2 ☐ 2<br>☐ 3 ☐ 3	☐ 0 ☐ 0<br>☐ 1 ☐ 1<br>☐ 2 ☐ 2<br>☐ 3 ☐ 3	☐ 1 Sim ☐ 1 Sim<br>☐ 2 Não ☐ 2 Não	☐ 0 ☐ 0<br>☐ 1 ☐ 1<br>☐ 2 ☐ 2<br>☐ 3 ☐ 3
**Exantema**	**Macular**	**Maculo-papular**	**Vesiculoso**
☐ 0 ☐ 0<br>☐ 1 ☐ 1<br>☐ 2 ☐ 2<br>☐ 3 ☐ 3	☐ 1 Sim ☐ 1 Sim<br>☐ 2 Não ☐ 2 Não	☐ 1 Sim ☐ 1 Sim<br>☐ 2 Não ☐ 2 Não	☐ 1 Sim ☐ 1 Sim<br>☐ 2 Não ☐ 2 Não
**Enantema**	**Hiper orofaringe**	**Adenomegalias**	**Cadeias ganglionares**
☐ 1 Sim ☐ 1 Sim<br>☐ 2 Não ☐ 2 Não	☐ 1 Sim ☐ 1 Sim<br>☐ 2 Não ☐ 2 Não	☐ 0 ☐ 0<br>☐ 1 ☐ 1<br>☐ 2 ☐ 2<br>☐ 3 ☐ 3	☐ 1 cervical<br>☐ 2 occiptal<br>☐ 3 supraclavicular<br>☐ 4 axilar<br>☐ 5 epitroclear<br>☐ 6 inguinal<br>☐ 9 NSA
**Hemorragias**	**Petéquias**	**Púrpura**	**Gengivorragia**
☐ 1 Sim ☐ 1 Sim<br>☐ 2 Não ☐ 2 Não	☐ 0 ☐ 0<br>☐ 1 ☐ 1<br>☐ 2 ☐ 2<br>☐ 3 ☐ 3	☐ 0 ☐ 0<br>☐ 1 ☐ 1<br>☐ 2 ☐ 2<br>☐ 3 ☐ 3	☐ 0 ☐ 0<br>☐ 1 ☐ 1<br>☐ 2 ☐ 2<br>☐ 3 ☐ 3

2629030035

Cod

Hepatomegalia
☐1 Sim ☐1 Sim
☐2 Não ☐2 Não

Dor percussão lombar unilateral
☐ 1 Sim ☐ 1 Sim
☐ 2 Não ☐ 2 Não

Esplenomegalia
☐0 ☐0
☐1 ☐1
☐2 ☐2
☐3 ☐3

Ascite
☐0 ☐0
☐1 ☐1
☐2 ☐2
☐3 ☐3

Dor abdominal à palpação
☐0 ☐0
☐1 ☐1
☐2 ☐2
☐3 ☐3

Rigidez de nuca
☐0 ☐0
☐1 ☐1
☐2 ☐2
☐3 ☐3

Estertores
☐0 Ausente
☐1 Unilateral
☐2 Unilateral difuso
☐3 Bilateral

Roncos
☐0 Ausente
☐1 Unilaterlal
☐2 Unilateral difuso
☐3 Bilateral

Sibilos
☐ 1 Ausente
☐2 Unilaterlal
☐3 Unilateral difuso
☐4 Bilateral

Sopro Cardíaco
☐0 ☐0
☐1 ☐1
☐2 ☐2
☐3 ☐3

Alterações Radiológicas
☐1 Condensacao alveolar
☐2 Infiltrado intersticial
☐3 Derrame pleural
☐4 Outros
☐5 aumento de área cardíaca

Diagnóstico

Diagnóstico Clínico
☐1 Dengue
☐2 Rubéola
☐3 Sarampo
☐4 Parvovirus B19
☐5 Mononucleose
☐6 Infec. aguda HIV
☐7 Varicela
☐8 Citomegalovírus
☐9 Enterovírus
☐10 Meningococcemia
☐11 Ricketsioses
☐12 Leptospirose
☐13 Febre amarela
☐14 Hepatites
☐15 Malária
☐16 Adenovírus
☐17 Rinovírus
☐18 meningites
☐19 Influenza
☐20 para-Influenza
☐21 USR
☐22 Outros

Diagnóstico por Síndromes
☐1 Exantemática
☐2 Linfoadenogálica
☐3 Hemorrágica
☐4 Ictérica
☐5 Respiratória
☐6 Neurológica
☐7 Febril
☐8 Diarreica
☐9 Missing

IgM-1
☐1 Positivo
☐2 Negativo

IgM-2
☐1 Positivo
☐2 Negativo

Isolamento
☐1 Positivo
☐2 Negativo

IgG -1
☐1 Positivo
☐2 Negativo

IgG-2
☐1 Positivo
☐2 Negativo

NS1
☐1 Positivo
☐2 Negativo

PCR
☐1 Positivo
☐2 Negativo

9390030037

Cod

Tabela II-Exame

Laboratório

Data Exame

Htc% Plaq.(x10³)

Hb Leucócitos

Bas Eos Jovens

Bast Seg

Linf Monócito Atípico

Tgo Tgp

Albumina FA

TAP(%) BbD

BbT Gama GT

Ureia Sódio

Creat

VHS Amilase

Glicose K+

Laboratório

Data Exame

Htc% Plaq.(x10³)

Hb Leucócitos

Bas Eos Jovens

Bast Seg

Linf Monócito Atípico

Tgo Tgp

Albumina FA

TAP(%) BbD

BbT Gama GT

Ureia Sódio

Creat

VHS Amilase

Glicose K+
